# Design and Test Research on Cutting Blade of Corn Harvester Based on Bionic Principle

**DOI:** 10.1155/2017/6953786

**Published:** 2017-10-22

**Authors:** Kunpeng Tian, Xianwang Li, Bin Zhang, Qiaomin Chen, Cheng Shen, Jicheng Huang

**Affiliations:** Nanjing Research Institute for Agricultural Mechanization, Ministry of Agriculture, Nanjing 210014, China

## Abstract

Existing corn harvester cutting blades have problems associated with large cutting resistance, high energy consumption, and poor cut quality. Using bionics principles, a bionic blade was designed by extracting the cutting tooth profile curve of the *B. horsfieldi* palate. Using a double-blade cutting device testing system, a single stalk cutting performance contrast test for corn stalks obtained at harvest time was carried out. Results show that bionic blades have superior performance, demonstrated by strong cutting ability and good cut quality. Using statistical analysis of two groups of cutting test data, the average cutting force and cutting energy of bionic blades and ordinary blades were obtained as 480.24 N and 551.31 N and 3.91 J and 4.38 J, respectively. Average maximum cutting force and cutting energy consumption for the bionic blade were reduced by 12.89% and 10.73%, respectively. Variance analysis showed that both blade types had a significant effect on maximum cutting energy and cutting energy required to cut a corn stalk. This demonstrates that bionic blades have better cutting force and energy consumption reduction performance than ordinary blades.

## 1. Introduction

In the recent years, minimizing energy consumption along with building a resource-saving and environment-friendly society has become a high priority in today's world. Also, agricultural processes have seen a fast pace of modernization, which have resulted in an increased mechanization of agricultural production. As a result, total power consumption of agricultural machinery has been rising, which in turn results in higher energy consumption of agricultural production equipment. As one of the world's major food crops, corn has been planted over large-areas across the world. Due to this, there is a requirement for a large number of corn harvesting machinery. Thus, minimizing the energy consumption of a corn harvester would significantly contribute to the reduction of carbon emissions of global agricultural equipment [1, 2]. A cutting blade is one of the key working parts of a corn harvester. As corn stalks are typically brawnier, the cutting process requires significantly large amounts of energy for corn harvesting, so small cutting resistance and low energy consumption requirements of the cutting blade are greatly beneficial and contribute to total energy consumption reduction for a corn harvester machine.

In present-day harvesters, two types of blades are used in corn harvesters, rotary type and reciprocating type. Among these two blade types, significantly higher proportion of research reported in literature has focused on rotary blades, where focus was on structural aspects and motion parameters of the blade [3, 4]. In the case of the reciprocating cutting blade, discussions focused primarily on blade arrangement and motion parameter matching [5, 6]. It is important to note that there is a lack of focus on blade structure parameters in this particular case. Currently, reciprocating cutting blades on the corn harvester use the triangle tip tooth type blades, which are widely used in rice and wheat harvesters. Using the rice and wheat harvester blade to cut corn stalks results in large cutting resistance, high energy consumption, and poor quality of stubble along with other issues. These issues can be attributed to the difference in the corn stalk from the rice and wheat stalk. Therefore, there is an urgent need to develop a new type of reciprocating cutting blades for cutting corn stalk.

Bionics as an emerging interdisciplinary field provides researchers with new research ideas and effective research methods in solving various problems including energy conservation and improving quality of cutting tools [7]. Insects and animals in nature through competition for survival have evolved over several million years. Evolution has resulted in excellent optimization of geometric structures and efficient energy utilization so that their physiological characteristics are highly adapted to the environment [8–10]. The application of animal physiology and morphology to agricultural machinery design through bionics has received considerable attention over the recent years. Bionics has been applied in the design of components of tools such as openers, plow, and stubble rotary tiller to allow drag reduction and reduce energy consumption. Qaisrani et al. [11, 12] and Soni and Salokhe [13, 14] applied bionic principles to the design of a bulldozer and ploughshare by using the convex hull structure of the mantis head and the beetle and designed the bionic bulldozer and bionic plowshares, respectively. A contrast test showed that the design based on bionic principles resulted in drag reduction in the range of 8% to 36%. Zhang et al. [15] applied structural characteristics of the rat claw to the design of a deep loosening shovel. This bionic shovel was designed using an exponential function curve. The contrast test showed that the bionic cultivation resistance of the deep loosening shovel was reduced by 8.5% to 39.5%. Similarly, based on the morphological characteristics of the mantis foot, a bionic cutting blade was designed by Li et al. [4, 16], which was demonstrated to lower both cutting force and energy consumption by 23% compared to an ordinary blade, and the effect of drag reduction was obvious.

Longicorn beetle called nature's “Sawyer” is a harmful pest commonly found on woody plants and grasses [17–19]. The unique curved tooth structure of the longicorn's palate has excellent chewing and cutting performance, which can easily crush or pinch stalks and branches of a plant. This often results in the plants becoming weak and often dying. In this paper, we focus on taking advantage of this problem. Using the palate of a common Longicorn beetle, *B. horsfieldi* (*Batocera horsfieldi*), as the bionic prototype and applying characteristics of *B. horsfieldi*'s palate to the design of cutting blades of a corn harvester, we focus on the development of a suitable cutting blade design for the corn harvester with low resistance, low energy consumption, and good quality stubble.

## 2. Bionic Blade Design

### 2.1. Analysis of *B. horsfieldi* Palate Structure and Morphology

**Figure 1:**
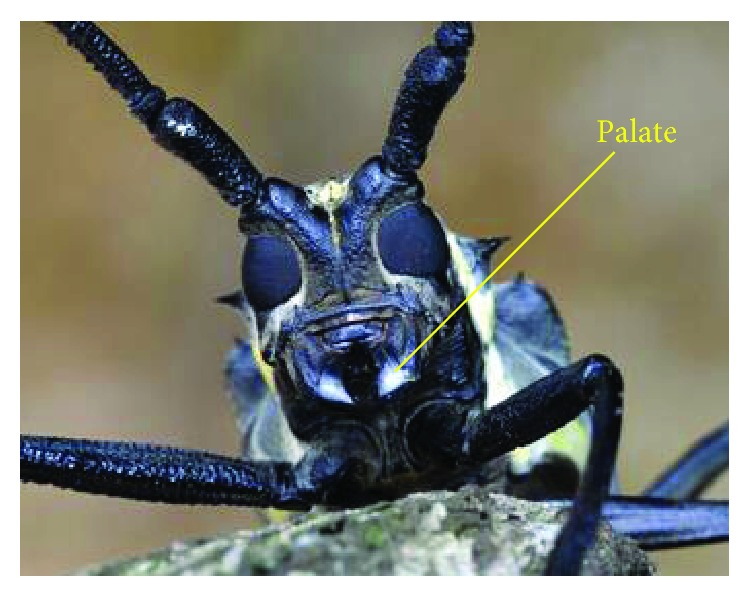
Typical specimen of *B. horsfieldi*.

**Figure 2:**
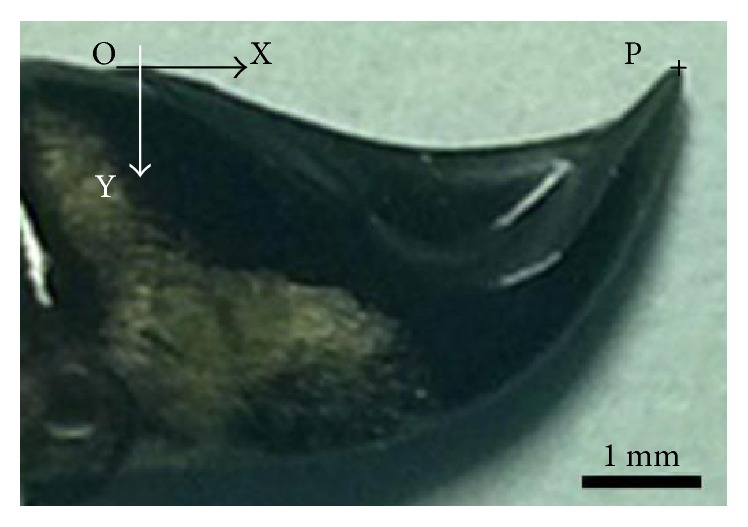
Magnified view of the *B. horsfieldi* palate.

*B. horsfieldi* (Figure 1) is typically 32 to 65 mm long, 9 to 20 mm wide, and has black or dark brown color. B. longicorn palate (Figure 2) is typically found to be 5 to 8 mm long, 3 to 5 mm wide with black semicrescent-shaped symmetrical morphology. The tooth edge (as shown in Figure 2, OP is tooth profile section) is sharp and surrounded by arc-shaped protrusions outside the maxilla to ensure that tooth edge has high strength. When eating, the left and right palates perform a simultaneous cutting movement directed towards the middle to cutoff the plant stalk. Kinematic characteristics of the palates are similar to the reciprocating double blades of a corn harvester in this paper, which is also the basis of this paper using the palate as a biomimetic object.

### 2.2. Contour Extraction of the Palate

**Figure 3:**
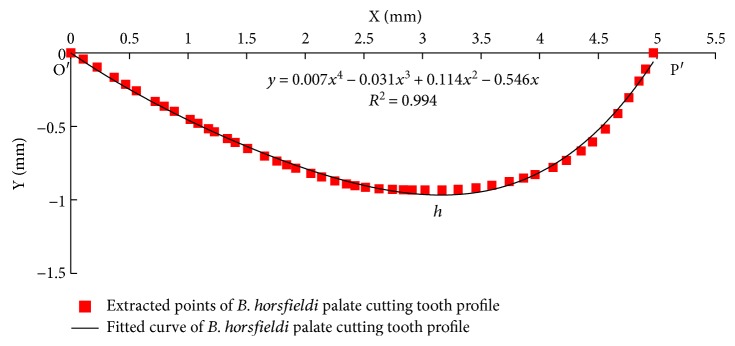
Extracted profile of *B. horsfieldi* palate (red) and the curve fit of the palate profile (black).

In Figure 2, O is the origin of the reference coordinate system, and OP is along the X-axis, a 2D plane exists along the Cartesian coordinate system XOY. Contour points of the palate were extracted using the Training Image Labeler image-processing tool in MATLAB (MathWorks, USA) [20]. Coordinates of the extracted boundary points are plotted by to extract the polynomial equation of the palate.  Figure 3 shows the extracted profile of the cutting parts and the fitting curve of the *B. horsfieldi* palate. The following equation is the palate fitting curve equation.

(1) $$\begin{array}{c} y=0.007x^{4}-0.031x^{3}+0.114x^{2}-0.546x, \end{array}$$

where 0 ≤ *x* ≤ 5.0 mm.

Coordinate points of O′ and P′ in Figure 3 correspond to points O and P in Figure 2. From the curve fit of the tooth profile shown in Figure 3, we can see that the length of the curved tooth of the palate O′P′ is 5.0 mm and palate height *h* is 0.9 mm. Correlation coefficient *R*^2^ of the fitted curve is 0.994, which indicated a good fit implying that the curve shape is highly similar to the actual contour curve of the palate.

### 2.3. Bionic Blade Design

**Figure 4:**
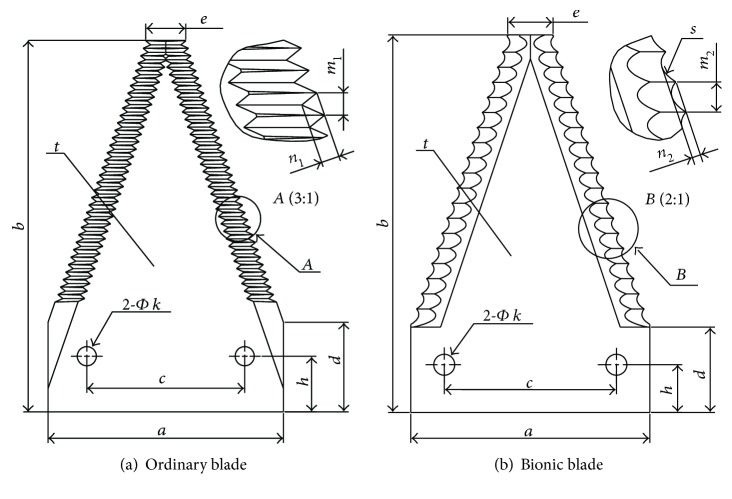
Structure of the cutting blade.

In order to facilitate comparative analysis, the bionic blade used the same substrate structural parameters as the ordinary rice and wheat harvester blade, that is, *a* = 76 mm, *b* = 120 mm, *c* = 51 mm, *d* = 29 mm, *e* = 13 mm, *h* = 18 mm, *k* = 6.2 mm, and *t* = 2.3 mm. The basic structural parameters of the blade substrate were used to build a 3D model of the bionic blade in PRO/E (PTC, USA). The spline-shaped palate profile was fitted using a spline curve, and the palate profile spline was evenly arranged on both sides of the blade. The tooth grooves on the blade edge were machined using a grinding die with the same shape as the palate profile. The direction of the groove was in parallel to the direction of the blade cutting motion to ensure that the cutting edge is sufficiently strong to minimize cutting resistance. Figures 4(a) and 4(b) show the structural diagram of the ordinary blade and bionic blade, respectively.

**Figure 5:**
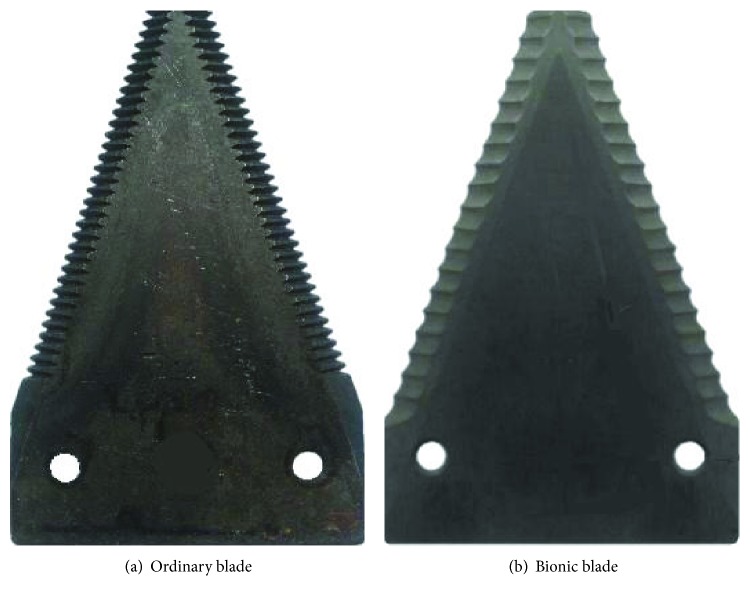
Images of fabricated cutting blades.

The difference between ordinary blade and bionic blade becomes apparent by comparing the two design, as shown in Figure 4. The difference is primarily due to the aspect of tooth profile, pitch, and height. In the case of an ordinary blade, the teeth are triangular in shape with a tooth pitch *m*_1_ of 2.6 mm and tooth height *n*_1_ of 2.0 mm. In contrast, the tooth profile of the bionic blade is a curved groove based on the curve fit “*s*” in Figure 4(b) of the *B. horsfieldi* palate profile. From the measurements of the palate shape, the bionic blade pitch *m*_2_ is 5.0 mm and tooth height *n*_2_ is 0.9 mm. Images of the actual fabricated ordinary blade and bionic blade are shown in Figures 5(a) and 5(b).

## 3. Cutting Test

The purpose of the test is to analyze the cutting performance of the bionic blade by comparing maximum cutting force required to cut a single stalk, cutting energy consumption, and cutting effect of the bionic blade against an ordinary blade.

### 3.1. Test Materials

Test materials were obtained from the National Corn Demonstration Base of Wucheng County, Shandong Province on October 9, 2016 (at harvest time). According to the corn harvester work requirements, corn stubble height requirement is approximately 10 cm with an average diameter of 20.5 mm. In order to facilitate quantitative comparative analysis, samples to be used as the test materials had a stubble height of more than 10 cm above the ground with a diameter of 20.5 ± 1 mm, grown well, and no pests on corn stalks. In addition, to maximize chances of maintaining both physical and mechanical properties of corn stalks during harvest along with eliminating the influence of water moisture content change on stalk material properties, experiments should be carried out within 48 h after stalk samples are collected and sealed.

### 3.2. Experimental Setup

**Figure 6:**
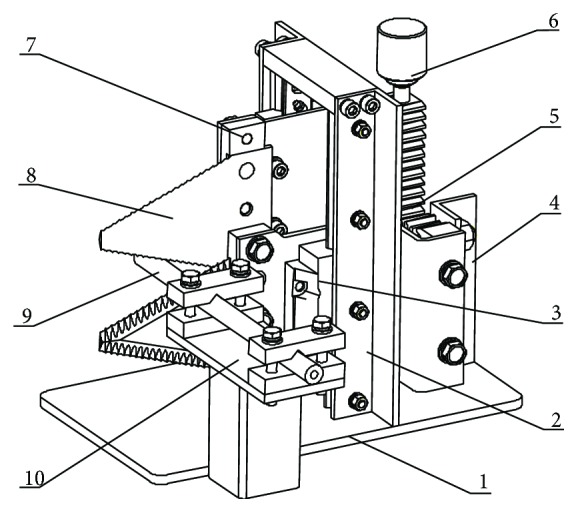
Schematic of the double-blade cutting device. (1) Baseplate, (2) support angle iron of sliding rail, (3) sliding rail, (4) gear fixed angle iron, (5) rack and pinion mechanism, (6) loading head, (7) blade fixed plate, (8) cutting blade, (9) corn stalk, and (10) stalk fixer.

A SUNS UTM6503 microcomputer control electronic universal testing machine was used to carry out the mechanical testing of the collected samples. A self-developed, double-blade cutting device is shown in Figure 6, which can be fitted with either an ordinary or bionic blade along with measurement tools such as a vernier caliper. The universal testing machine used in this study has a measuring range of 100 N~5 KN, with an accuracy grade of 0.5, and a loading speed adjustment range of 0.001~500 mm/min, and the speed relative error rate is within ±1.0%.

**Figure 7:**
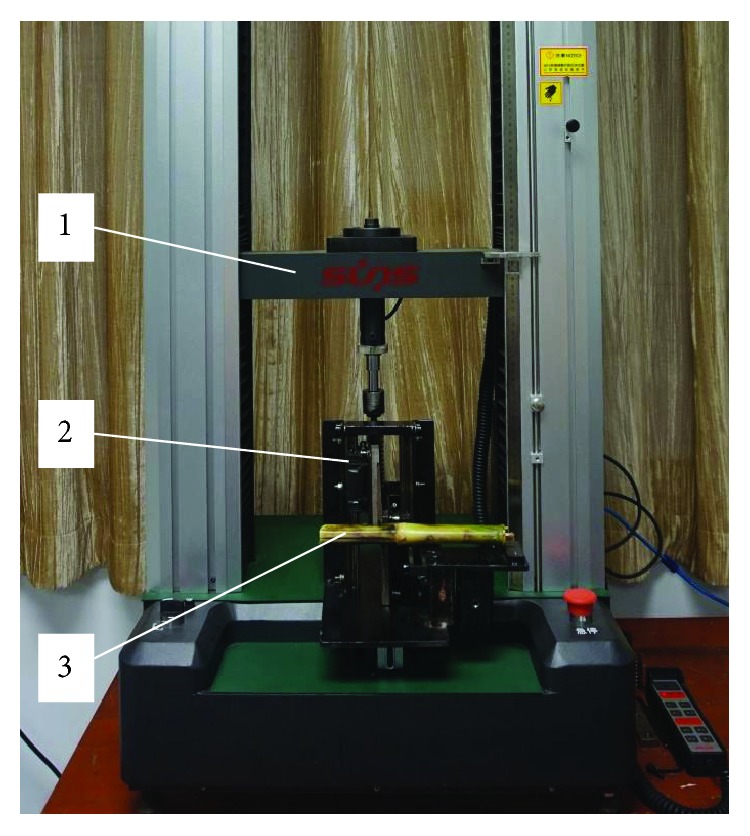
Experimental setup with the double-blade cutting force testing system. (1) Electronic universal testing machine, (2) double-blade cutting device, and (3) corn stalk.

During the test, the loading head of the double-blade cutting device was connected with the driving head of the universal testing machine to form a double-blade cutting force testing system, as shown in Figure 7. Loading the universal testing machine at a certain speed resulted in the rack and pinion drive mechanism of the double-blade cutting device engaging in the meshing movement. In turn, the meshing movement of the rack and pinon drives the blades fixed on the fixed plate at the same speed but in the opposite direction. This allows the cut to be made for the corn stalk fixed on the stalk fixing plate. Cutting force and displacement measurements are made through the sensors attached to the universal testing machine measurement system, and a data acquisition system attached to a computer dynamically records the corresponding change over time.

### 3.3. Test Method

The single-factor test was used to cut individual corn stalks with both the ordinary and bionic blades. Each test was repeated 20 times. The speed of the universal testing machine was set at 25 mm/min. After cutting, stubbles were sorted by category based on the cutting blade used, so as to compare the cutting effect of the two blades.

**Figure 8:**
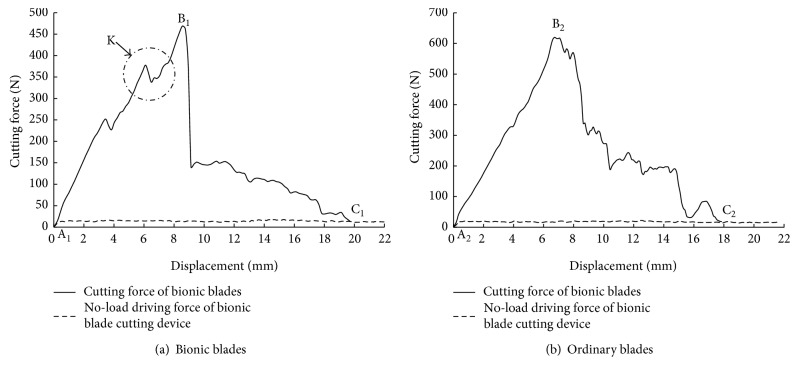
Typical cutting force-displacement curves of different types of blades.

Cutting energy consumption, that is, energy consumption of the blade required to cut the stalk is calculated by the difference between total energy consumption of the cutting force and energy consumption of the driving force at no load. Cutting force-displacement curve and no-load driving force-displacement curves were plotted. Area under these curves was calculated using numerical integration to obtain the energy required. In Figure 8, the area between the cutting force curve and no load curve is the cutting energy. Hence, cutting energy consumption can be given as

(2) $$\begin{array}{c} W=\overset{n}{\underset{i=1}{\sum }}F_{i}\cdot \Delta x-\overset{n}{\underset{i=1}{\sum }}F_{i0}\cdot \Delta x, \end{array}$$

where *W* is cutting energy, *F_i_* is cutting force value corresponding to the *i*th displacement interval, *F*_*i*0_ is no-load driving force value corresponding to *i*th displacement interval, and ∆*x* is loading displacement of universal testing machine data recording frequency interval.

### 3.4. Test Results and Analysis

**Figure 9:**
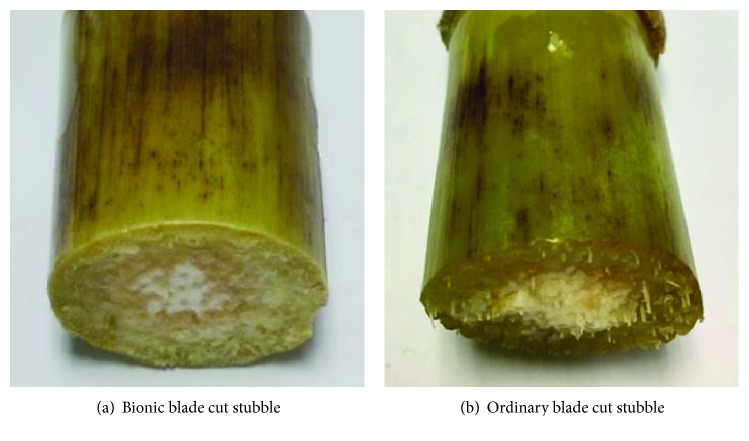
Comparison of cut stubble using both bionic and ordinary blades.

Equipped with ordinary and bionic blades, a double-blade cutting device for a single stalk, typical cutting force-displacement curves and no-load driving force-displacement curves for both of blades are shown in Figure 8. Solid lines are used in the figure to depict the cutting force-displacement curves of the bionic and ordinary blades, respectively. Dotted line represents no-load driving force-displacement curve of the cutting device equipped with ordinary and bionic blades. Cutting effects of both blades are shown in Figure 9. Maximum cutting force and cutting energy consumption of different blades are shown in Table 1. One-way ANOVA was used to analyze factors that affect maximum cutting force and energy consumption. The variance analysis table is shown in Table 2.

Based on Figure 8, cutting force-displacement curves for the two blades can be divided into two phases, namely, squeeze and cutting phase.

Cutting force was gradually increased from zero to the maximum value as indicated by the curve segments AB_1_ and AB_2,_ shown in Figure 8, this is the squeeze phase. From the cutting force-displacement curve of the bionic blade, we can see that the cutting force in this phase was accompanied by a serrated sudden drop in the magnitude of force with an overall general increasing trend in the magnitude(as shown by circle K). This is primarily due to the simultaneous cut made by the upper and lower blade to the corn stalk. Bionic blades have larger tooth pitches and sharper tips. When bionic blades come into contact with the corn stalk, unit area stress on the stalk is large, which results in local cutting phenomenon in the squeeze phase occurring at the tooth tip. Whereas, for ordinary blades, because of the close arrangement of cutting teeth, unit area stress of corn stalk is lower for the same cutting driving force. As a result, in comparison to the bionic blade, an ordinary blade shows a more prominent squeeze effect on the corn stalk. Comparison of the cutting force curve in the squeeze phase reveals that the bionic cutting blade has an advantage over the ordinary blade and facilitates ease of cut.

Cutting force decreases from the maximum value down to a stabilization phase, as shown by B_1_C_1_ and B_2_C_2_ in Figure 8. This is the stalk cutting phase. Here, from the cutting force-displacement curves, it can be seen that when cutting with a bionic blade, cutting force drastically drops from the maximum value ranging from 374.20 N to 526.85 N to lower values between 117.63 N and 188.50 N. Whereas, for the ordinary blade, a gradual decrease in cutting force can be observed. This difference can be attributed to the unique curved teeth and tooth back groove structure of the bionic blade. First, after the partial cleave at the tooth tip during the squeeze phase, due to the existence of the arc-shaped cutting edge structure, the tooth edge in the subsequent cutting process is undergoing slip cutting. Existence of the slip cutting action greatly reduces cutting resistance, thereby improving the cutting effect. Second, adjacent protrusions of the arc tooth boundary form a sharp voussoir structure, under the driving force of cutting. Here, the sharp wedge allows transversal splitting of the stalk. However, because in the case of the ordinary blade, close arrangement of triangular teeth and due poor slipping, the structure of ordinary rice and wheat harvester blades hindered cutting of corn stalks, resulting in increased cutting resistance.

A comparison of cut stubble effect is shown in Figure 9. It can be seen that compared with the ordinary blades, bionic blades can cut the corn stalk more effectively, that is, no uncut fiber was seen and cutting stubble has a higher uniformity. This implies that cutting quality using a bionic blade is better compared to the ordinary blade used here.

According to the results in Table 1, for bionic blades, mean value of maximum cutting force of a single stalk is 480.24 N and standard deviation is 37.83 N, and mean value of cutting energy is 3.91 J and standard deviation is 0.46 J. For ordinary blades, mean value of maximum cutting force of a single stalk is 551.31 N and standard deviation is 68.81 N, and mean value of the cutting energy is 4.38 J and standard deviation is 0.45 J. Results show that average cutting force and cutting energy are reduced by 12.89% and 10.73%, respectively, for the bionic blade. This indicates that the bionic blade has the advantage to reduce both cutting force and energy consumption.

From the variance analysis in Table 2, *F*-test was carried out on maximum cutting force data and cutting energy data for different blades at the significance level *α* = 0.05. *F* value is 12.2856 and 7.8463 (*F* < *F*-crit), and the significance level *P* value is 0.0016 and 0.0091 (*P* < 0.01), respectively, which indicates that different types of blades have a very significant impact on the maximum cutting force and cutting energy of a single stalk.

## 4. Conclusion

1. Using bionic principles with *B. horsfieldi* palate as a bionic prototype, a corn harvester bionic cutting blade is designed to solve problems associated with existing corn harvester blades such as large cutting resistance, high energy consumption, and poor cut quality.
2. In order to verify the cutting performance of the bionic blades, a double-blade cutting force test system was used to compare the performance of bionic and ordinary blades to cut corn stalks. Experimental results show that the bionic blade has an advantage as it facilitates easier cut than the ordinary blade, and cut stubble is more uniform. This demonstrated that the bionic blade's cutting quality is significantly better.
3. Mean value and one-way ANOVA were used to statistically analyze the obtained data for average maximum cutting force and cutting energy consumption of both bionic and ordinary blades, which are 480.24 N and 551.31 N, and 3.91 J and 4.38 J, respectively. Compared to ordinary blades, average cutting force and cutting energy were reduced by 12.89% and 10.73%, respectively.

## Figures and Tables

**Table 1:** Single stalk maximum cutting force and cutting energy consumption test results for the bionic and ordinary blade.

Blade type	Test number	The maximum cutting force (N)	Cutting energy (J)
Bionic blade	1	516.19	4.37
	2	526.85	4.39
	3	489.30	4.16
	4	499.32	3.73
	5	477.13	4.09
	6	374.20	2.92
	7	483.11	4.21
	8	476.99	4.02
	9	454.20	3.31
	10	491.13	4.29
	11	484.51	3.44
	12	450.00	3.39
	13	519.22	4.46
	14	509.07	4.12
	15	452.43	3.76
	Mean	480.24	3.91
	Std. deviation	37.83	0.46

Ordinary blade	1	429.32	3.64
	2	540.28	4.28
	3	493.39	3.96
	4	524.88	4.71
	5	491.65	4.10
	6	632.57	5.06
	7	504.92	4.18
	8	676.97	4.97
	9	551.31	4.54
	10	605.31	4.52
	11	570.27	4.15
	12	510.13	4.29
	13	601.23	5.03
	14	494.34	3.65
	15	643.03	4.63
	Mean	551.31	4.38
	Std. deviation	68.81	0.45

**Table 2:** Variance analysis table.

Test index	Sources of variation	Sum of squares	Degrees of freedom	Mean square	*F* value	*P* value	*F*-crit
The maximum cutting force	SS_A_	37,874	1	37,875	12.2856	0.0016	4.1960
	SS_E_	86,320	28	3083			
	SS_T_	124,195	29				

Cutting energy	SS_A_	1.6568	1	1.6568	7.8463	0.0091	4.1960
	SS_E_	5.9122	28	0.2111			
	SS_T_	7.5689	29				

SS_A_: sum of squares for factor; SS_E_: sum of squares for error; SS_T_: sum of squares for total.
